# How working-age population education and health of older people shape the burden of population aging: A comparative study of Macau, Hong Kong, and Singapore

**DOI:** 10.3389/fpubh.2022.1031229

**Published:** 2022-11-03

**Authors:** Dong-mei Xue, Qian Bai, Ying Bian

**Affiliations:** ^1^Institute of Chinese Medical Sciences, University of Macau, Taipa, Macau SAR, China; ^2^State Key Laboratory of Quality Research in Chinese Medicine, University of Macau, Taipa, Macau SAR, China; ^3^Department of Public Health and Medicinal Administration, Faculty of Health Sciences, University of Macau, Taipa, Macau SAR, China

**Keywords:** population aging, educational attainment, working-age population, older people, burden, Macau, Hong Kong, Singapore

## Abstract

Macau, Hong Kong and Singapore are all facing increasing population aging. Those aged 65 and over make up the old-age population. The working-age population refers to the population aged 15 to 64. Conventionally, the burden of population aging is measured by the Old-Age Dependency Ratio, which is the ratio of the old-age population to the working-age population. As life expectancy rises, depending exclusively on age to calculate the burden of aging hinders the development of effective anti-aging strategies. The working-age population's education and the elderly's health affect the aging burden's support and generator, respectively. Including them in the calculation gives us a fuller view of the burden of aging.

**Objective:**

To compare the population aging burden in Macau, Hong Kong, and Singapore by including working-age population education and elderly health.

**Methods:**

The overall, working-age and old-age population and proportion, as well as the Old-Age Dependency Ratio of Macau, Hong Kong, and Singapore, were collected from the World Bank database. The life expectancy at 65 was extracted from the 2022 World Population Prospect. The tertiary education rate of the working-age population and the self-rated health status of the old-age population were retrieved from governments' statistical reports. We then calculated the Education-Health Adjusted Old-Age Dependency Ratio, a set of four equations showing the support of the working-age population on the old-age population, where OADR_h_t_ and OADR_uh_t_ represent the burden of healthy and unhealthy old-age population on the working-age population with tertiary education; similarly, OADR_h_nt_ and OADR_uh_nt_ indicate the burden placed on the working-age population without tertiary education by healthy and unhealthy old-age population. Lastly, for comparison with the conventional Old-Age Dependency Ratio, we generated the Weighted Education-Health Adjusted Old-Age Dependency Ratio.

**Results:**

Hong Kong has the greatest old-age population proportion and Old-Age Dependency Ratio, yet its growth rates are moderate and stable, ranging from 0 to 4% and 0 to 6%, respectively. Macau and Singapore experienced sharper changes in old-age population proportion and the Old-Age Dependency Ratio, with Macau's Old-Age Dependency Ratio varying between −2.66 and 8.50% and Singapore's ranging from −1.53 to 9.70%. Three cities showed different patterns in four Education-Health Adjusted Old-Age Dependency Ratio indicators. In Macau, the OADR_h_nt_ and OADR_uh_nt_ increased by 0.4 and 6.2, while the OADR_h_t_ and OADR_uh_t_ decreased by 13.5 and 15.3 from 2004 to 2016. In Hong Kong, only the OADR_uh_t_ fell by 9.4, and the other three increased from 2003 to 2015. In Singapore, the OADR_h_nt_ and OADR_h_t_ increased by 3.8 and 1.0, while OADR_uh_nt_ and OADR_uh_t_ decreased by 1.2 and 3.9 from 2007 to 2011. The Weighted Education-Health Adjusted Old-Age Dependency Ratios are all smaller than the conventional Old-Age Dependency Ratio in the three regions, particularly in Singapore. The Weighted Education-Health Adjusted Old-Age Dependency Ratio of Singapore was reduced by 9.5 to 30.5% compared with the conventional Old-Age Dependency Ratio, that of Hong Kong reduced by 6.2 to 22.5%, and that of Macau reduced by 4.4 to 16.1%.

**Conclusion:**

This is the first study to compare the aging burden in Macau, Hong Kong, and Singapore in connection to working-age population education and elderly health. With the new assessment, the burden of population aging in three regions has been reduced, showing that improving the education of the working-age population and maintaining older people's wellbeing can assist authorities to deal with population aging, especially in Macau and Hong Kong.

## Introduction

Population aging has become a worldwide problem. Due to increased longevity, declining fertility, and the aging of “baby boom” generations, as estimated, the old-age population (aged 65 and over) is expected to grow from 9.3% in 2020 to 16.0% in 2050 (1, 2). This shift in population structure brings enormous societal and financial challenges to all nations, including declining saving rates, labor shortages, deteriorating government finances due to increased spending on elderly-related items, and reduced innovativeness (3, 4). It is important to estimate the burden of aging for the purpose of developing effective economic and labor policies to deal with population aging.

The old-age population proportion and the Old-Age Dependency Ratio (OADR) are two widely used indices that reflect the social burden of the aging population on society (5). The former means the percentage of the old-age population of the total population, and the latter is the ratio of old-age population to the working-age population (aged 15 to 64) (6). Although these demographic parameters are straightforward, they only take into account chronological age (7), therefore providing a limited picture of population aging (8). Growing OADRs in most countries paint a gloomy picture of an aging society's future (9, 10). Notably, the OADR neglects age-specific characteristics which change over time and place, and conclusions drawn from it may be exaggerated (8). Nowadays, people over 65 are healthier due to higher life expectancies, and the meaning of chronological age changes as longevity increases (11, 12). When considering other factors such as education and health status, the burden of the population aging may not be as disastrous.

Driven by marriage postponement, low fertility, and cohabitation, some industrialized and urbanized Asian countries or regions have joined the second demographic transition, resulting in an aging society (13, 14). Located in East Asia, Macau, Hong Kong, and Singapore share many social and economic similarities (Table 1) (15, 16). Moreover, they are all confronted with essential issues related to an aging society. In 1983, Hong Kong became an aging society (over 7% of the population is older people) and in 2013, it transitioned to an aged society (over 14% of the population is elderly) (17). Before 1980, Macau was once an aging society, but young mainland Chinese immigrants arrived in the late 1980s, resulting in a much younger age structure. However, in 1994, Macau's old-age population rebounded to over 7% and remained an aging society for decades (18). Population aging in Hong Kong and Macau will further intensify, with the old-age population proportion in Macau reaching 22.4% in 2031 (18); and one-third of Hong Kongers will be 65 or older by 2040 (18). Singapore entered an aging society in 2004, roughly 20 years after Hong Kong and 10 years after Macau (17). Changes in different areas have been made to mitigate Singapore's rapid aging (19). Meanwhile, the level of education and medical care are different in the three cities (20). Combining education and health factors can help understand the burden of aging in different regions.

**Table 1:** Basic information about Macau, Hong Kong, and Singapore.

	**Macau**	**Hong Kong**	**Singapore**
Colony	Portugal (1557–1999)	UK (1842–1997)	UK (1819–1942)
Duration as a colony (year)	442	155	123
Sovereignty	SAR of China	SAR of China	Independent
Major ethnic group	Chinese	Chinese	Chinese
Land area (sq. km)	33.6	1,106.4	728.6
Population density (People per sq. km) (2021)	20,012	7,060	7,692
Total fertility rate (Births per woman) (2020)	1.2	0.9	1.1
Life expectancy at birth (Year) (2020)	84	85	84
Human Development Index	Very high	Very high	Very high
Year of entering an aging society	1,994	1,983	2,004
Population aged 65 and older and its proportion (2021)	83,821 (12.7%)	1,401,410 (18.9%)	777,976 (14.3%)

To date, there are no studies comparing the aging burden in Macau, Hong Kong, and Singapore that combine health and education. To fill this research gap and provide new insights for the authorities to cope with population aging, first, we collected the proportion of 65-and-older people and the OADR to assess population aging from a conventional perspective. Second, to understand the education and health levels in the three regions, we compared the educational attainment of working-age people and the life expectancy at age 65. Lastly, we adopted the Education-Health Adjusted Old-Age Dependency Ratio (EHA-OADR) and Weighted Education-Health Adjusted Old-Age Dependency Ratio (WEHA-OADR) to explore how education and health affect the population aging burden.

## Literature review

Population aging has a negative economic impact on labor markets, public pension systems, and long-term care for older people, according to many studies. First, workforce size and quality affect economic growth. The labor force participation of adults 50 and beyond is declining, as is their stock of assets. Aging societies face weak or stagnant economic growth due to a tight labor market and dissaving (21). Second, aging populations make pension planning challenging. Declining contributors and rising beneficiaries threaten publicly financed pay-as-you-go pensions, while fully funded systems take longer to offer substantial pensions (2). Lastly, declining birth rates lead to a scarcity of caretakers for older persons, increasing the demand on society to support the elderly (22).

Scholars once assumed that because illnesses increase with age, the elderly need more health care, thus bring burden to the society. To address the problem of aging burden, most research focused on the Old-Age Dependency Ratio (OADR). The dependency ratio was first introduced in 1913 by Carl Ballod (23) and has been adopted in research about the demographic dividend, which is considered as represent the ratio of non-working to working people (24–26). The OADR has been widely used since the 1970s, as older people have usually retired from economically productive work, they must depend on the working population to produce the resources needed. The size of the assumed retired population compared to the assumed working population approximates the aged dependency (27). Recent studies have shed light on the impact of population aging burden in different countries. B Ingham et al. anticipated and analyzed the OADR of the UK and Latvia and stated that while these two nations will have similar OADR by 2050, there are major cultural, skills, and pension system variations (10). MT Uddin et al. studied OADR in Bangladesh from 1951 to 2011 and observed an upward trend (28). Stepan evaluated the influence of population aging on economic dependency ratio in the Czech Republic and Slovakia. The decomposition of the indicator suggests a large old-age cohort contribution, which puts pressure on fiscal stability (29).

However, studies have shown that age is not the primary factors that influences the burden of population aging, but the socioeconomic factors and cost management (30, 31). Age-specific health gains and medical technology advancements reduce aging-related health care expense (32), which means we shouldn't assess population aging burden by age alone, but by social context. Much of literature emphasizes the effect of education and health on population aging. It's considered that better-educated civilizations have more resources for elderly support (12). And the age at which a person is considered elderly should take into account the improvement or deterioration of the individual's health (33). For one thing, increased educational attainment can compensate for the negative effects of population aging, thereby increasing society's capacity to provide upward intergeneration transfer for older adults (34). Some evidence also suggests that improvements in human capital can offset reductions in the size of working-age population (35–37), implying that enhancing the education of labor force can help cope with the declining size of working-age population. For another, as older people's health improves, the burden of population aging lessens (38). Existing research reveals that elderly people's life expectancy has grown due to better material conditions, fewer infectious diseases, and more pension funds and health care (39). While it has often been assumed that as age increases, so does the cost of elderly care (40). Further study confirms that the last year of life is the greater driver of elderly health care spending, and that the cost is higher for those who die younger (41) than for those who die older (42). Therefore, increasing life expectancy reduces older people's health care costs.

To provide a broader picture of population aging, Sanderson and Scherbov first proposed the characteristics approach to measure population aging. This approach includes age-related socioeconomic factors such as life expectancy and mortality rate. It is beneficial to trigger new ideas on age-related policy making (8). They then proposed several alternative measures to compare with the conventional dependency ratio (43). One of these alternative ratios is the ratio of non-workers to workers weighted by the labor force participation rates, which considers anyone not in the labor force as a dependent in old-age dependency. Another measure, to illustrate intergenerational transfers, calculates the ratio of consumers to producers based on consumption and income weights. Tilak Abeysinghe proposed a savings-adjusted dependency ratio that aims to preserve the exogenous characteristics of the traditional demographic ratio and to make short term projections of savings. The results for Japan and Singapore suggest that the conventional OADR greatly exaggerates the social burden imposed by older people (44). Researchers in Asia used the characteristics approach to study aging, which provides new ideas to tackle population aging in Asia. Inspired by the characteristics approach, Kye introduced the Education-Health Adjusted Old-Age Dependency Ratio (EHA-OADR), an alternative index of population aging burden that incorporates working-age population education and older people's health. Educational attainment of working-age people and elderly health affect the capacity and burdens of population aging, respectively (12). Kye calculated the EHA-OADR for Korea from 2000-2010 and compared the Weighted Education-Health Adjusted Old-Age Dependency Ratio (WEHA-OADR) with the conventional OADR, showing that the alternative index grows more slowly (12). Xiong et al. (45) followed Kye's research and compared aging in China, Japan, and South Korea using EHA-OADR and WEHA-OADR. They concluded that traditional OADR may underestimate aging in developing countries (46).

However, there is still a lack of research on the burden of population aging in Macau, Hong Kong, and Singapore that combines working-age population education and the health of older people. To see whether the alternative indices are different from the conventional OADR, we proposed three research questions:

Q1: Does the trend of EHA-OADR vary among the three cities?
Q2: Is the aging burden assessed by WEHA-OADR less than that of conventional OADR? Or vice versa?
Q3: Can we conclude that the longer region stays in an aging society, the greater the disparity between WEHA-OADR and conventional OADR?

## Methods

### Data sources

The overall, old-age, working-age populations, proportions, and the conventional OADRs since the first year of entering an aging society of Macau, Hong Kong and Singapore were retrieved from World Bank database (https://data.worldbank.org/). Next, we obtained statistics on the educational attainment of working-age people from 2001 to 2021. Macau data were collected from the annual Employment Survey (34); Hong Kong data from the General Household Survey (35); and Singapore data from the Department of Manpower Research and Statistics (36). Then, life expectancy at 65 in the past two decades as a proxy for regional health levels were extracted from the 2022 World Population Prospect (https://population.un.org/wpp/).

To calculate EHA-OADR and WEHA-OADR, we also needed the proportion of healthy and unhealthy older people, which life expectancy cannot directly provide. We preferred self-rated health as an indicator of the health status of older people. This item asks respondents to rate their general health on a 3- to 5- point scale. Self-rated health is a popular health indicator due to its feasibility, reliability, and ability to predict mortality (47, 48). We obtained results on the self-rated health of older people in Macau in 2004 and 2016. The former was from the Report on the Long-Term Care Needs of the Elderly in Macau (49), and the latter was from the Macau Health Survey 2016 (50). For Hong Kong, we gained data from the Report on Population Health Survey in 2004 and 2015 (31). For Singapore, we collected data from the 2007 National Health Surveillance Survey (51) and the 2011 National Survey of Senior Citizens (52). We defined “good, very good and extremely good” as “healthy” and “fair, poor, and very poor” as “unhealthy”. Because the data in the three regions were not collected on a regular basis, we were unable to obtain self-assessed health data for Macau, Hong Kong, and Singapore over the same years. We elaborated on this in the discussion section.

### Calculation of EHA-OADR and WEHA-OADR

Education and health are essential indicators of population heterogeneity and socioeconomic development (53). The EHA-OADR is an alternative index that incorporates education and health, thus providing a more comprehensive picture of the burden of aging on society. According to Kye's description (12), we divided working-age people by tertiary education and older people by health. Using this classification, we can calculate four EHA-OADR formulas as follows.


(1) $$\begin{array}{lll} OADR_{h\text{\_}nt} & = & \frac{E_{h}}{WK_{nt}} \end{array}$$

(2) $$\begin{array}{lll} OADR_{h\text{\_}t} & = & \frac{E_{h}}{WK_{t}} \end{array}$$

(3) $$\begin{array}{lll} OADR_{uh\text{\_}nt} & = & \frac{E_{uh}}{WK_{nt}} \end{array}$$

(4) $$\begin{array}{lll} OADR_{uh\text{\_}t} & = & \frac{E_{uh}}{WK_{t}} \end{array}$$

where, E_h_ indicates number of healthy older people, E_uh_ represents number of unhealthy older people, WK_nt_ is the number of non-tertiary-educated working-age people and WK_t_ represents the number of tertiary-educated working-age people.

The above equations measure the number of healthy and unhealthy older people per working-age person in a given education category. However, it is difficult to directly compare these indices with the conventional OADR. Theoretically, as the number of working-age people with tertiary education increases, the OADR_h_t_ and OADR_uh_t_ may decrease as the denominator becomes larger. As the elderly health status improves, the numerators of OADR_uh_t_ and OADR_uh_nt_ become smaller. But the total number of older people also increases, suggesting that the number of unhealthy older people may not decrease. To make comparison to the conventional OADR, the four equations are combined and weighted into the WEHA-OADR. The WEHA-OADR formula is defined as follows.


(5) $$\begin{array}{l} WEHA-OADR=\frac{W_{h}\times E_{h}+W_{uh}\times E_{uh}}{W_{nt}\times WK_{nt}+W_{t}\times WK_{t}} \end{array}$$

where W_h_ means the assigned weight to E_h_, W_uh_ indicates assigned weight to E_uh_, W_nt_ is assigned weight to WK_nt_, and W_t_ represents assigned weight to WK_t_.

The WEHA-OADR derives from two assumptions: at the societal level, (1) healthy older people place a smaller burden on society than unhealthy ones (W_h_≤W_uh_), and (2) working-age people with tertiary education provide more support than their counterparts without tertiary education (W_nt_≤W_t_). When there is no difference in the social burden due to the health status of the elderly and the support due to the educational attainment of working-age people (W_h_ = W_uh_ and W_t_ = W_nt_), then WEHA-OADR is equal to conventional OADR. Therefore, the setting of both weights should meet the equation W_t_ + W_h_ = W_nt_ + W_uh_.

There are two ways to set the weights. First, we can set weights based on empirical data. The investigated health expenditure for healthy and unhealthy older population can be used to determine W_h_ and W_uh_. Similarly, W_t_ and W_nt_ could be identified by measuring the different income between the working-age people with or without tertiary education. Second, we can use hypothetical weights. For example, we assume that healthy older people bring 10% less burden to society than unhealthy older people, then W_uh_ is set to 1.0 and the W_h_ to 0.9. We also need to consider W_t_ + W_h_ = W_nt_ + W_uh_. Therefore, we set the weight of the support capacity of working-age people with tertiary education (W_t_) at 1.1 and that of working-age people without tertiary education (W_nt_) at 1.0. We chose the hypothetical calculation because it can demonstrate how senior health and the educational attainment of working-age people influence the structure of old-age dependency. Three different weights of 10, 20, and 30% were set. Data were shown as the proportion of dependents per 100 working-age people.

## Results

### Conventional methods for measuring population aging and its burden

Conventionally, the process of an aging society is measured by the old-age population proportion, which means the percentage of the old-age population of the total population. A society in which the old-age population accounts for more than 7% of the total population is called an aging society, and when it exceeds 14%, it is defined as an aged society. The burden of population aging is often assessed by the Old-Age Dependency Ratio (OADR), which is the ratio of the old-age population to the working-age population.

**Figure 1:**
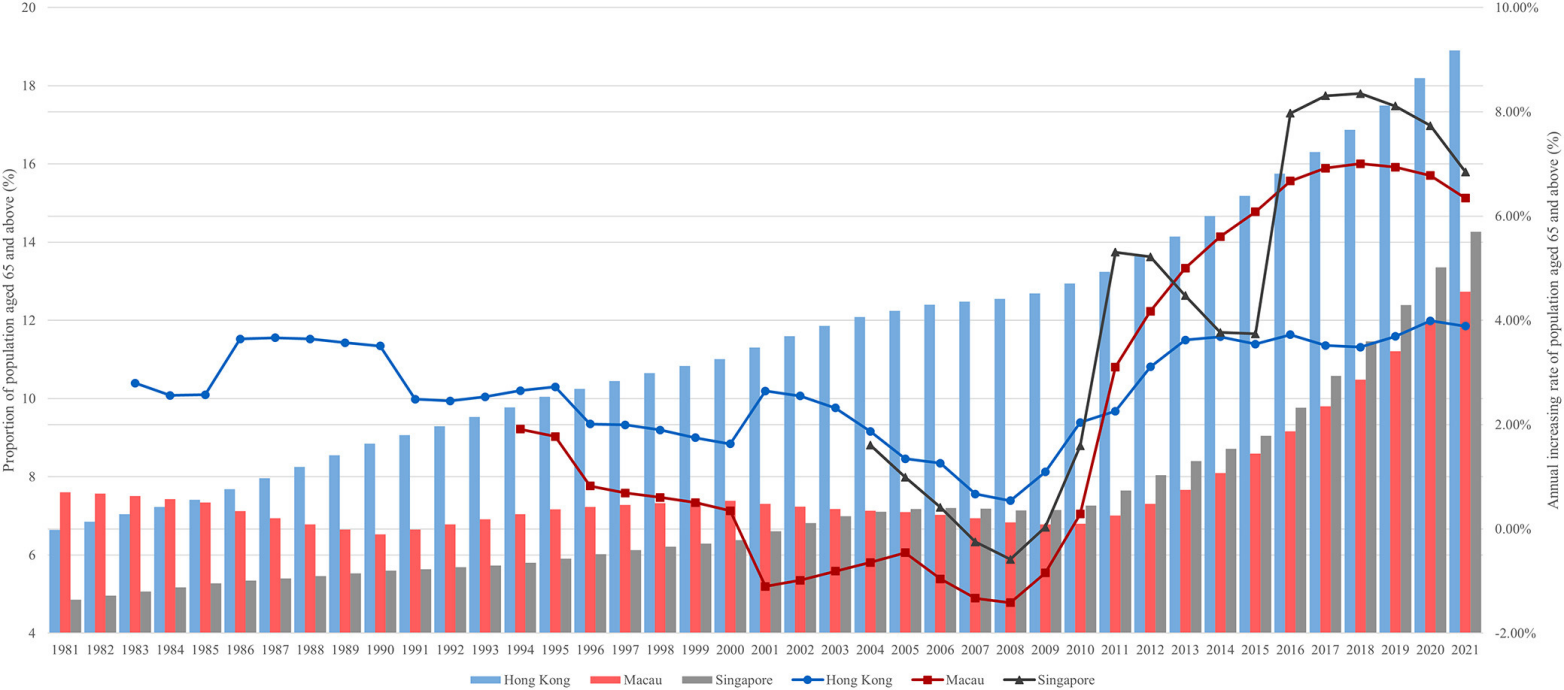
Proportion of 65+ population and its annual increasing rate since entering an aging society.

Old-age population proportion in Hong Kong was much higher than in Macau and Singapore, but the latter two grew at a faster rate. Hong Kong, Macau, and Singapore entered the aging society in 1983, 1994, and 2004 (Figure 1). Moreover, Hong Kong and Singapore transitioned to an aged society in 2013 and 2021. In 2021, the Old-Age Population Proportion in Hong Kong, Macau, and Singapore was 18.90, 12.73, and 14.27%, respectively. Hong Kong had a much higher proportion of older people than Macau and Singapore between 1985 and 2021. Between 2001 and 2017, the proportion of older people in Hong Kong reached more than 1.5 times that of the other two cities. Although the growth rates of the three regions were different, they all reached their lowest point in 2008 and then rebounded. During the statistical period, the growth rate of the old-age population in Hong Kong had always maintained between 0 and 4%. Macau and Singapore reached their highest growth rates of 7.00 and 8.35% in 2018.

**Figure 2:**
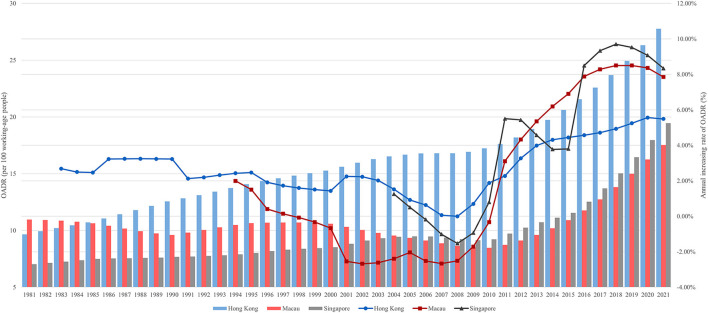
OADR and its annual increasing rate since entering an aging society.

Trends in OADR in Hong Kong, Macau, and Singapore were consistent with trends in the old-age population proportion (Figure 2). In 2021, the OADR for Hong Kong, Macau, and Singapore was 27.8, 17.5, and 19.5, suggesting that Hong Kong had a heavier aging burden than the other two cities. Since 1985, Hong Kong's OADR has been larger than the other two regions. In 2010, every 100 Hong Kong working-age people had to support 17.2 older people, compared to 8.5 in Macau and 9.2 in Singapore. The growth rate of OADR in Hong Kong, Macau, and Singapore all reached their lowest levels around 2008 and then peaked around 2018. Notably, after entering the aging society, Macau's OADR dropped from 10.7 in 1997 to 8.5 in 2010, and Singapore's declined from 9.5 in 2005 to 8.5 in 2009. However, after the end of descending growth rate, both Macau and Singapore saw a rebound in OADR growth, with Macau's peak at 8.50% and Singapore's peak at 9.70% in 2018. The growth rate of OADR in Hong Kong, meanwhile, has remained positive since 1983, rising from 2% to nearly 6% in the last decade.

### The educational attainment of working-age population and health status of the elderly population

Before combining them to compute the social burden of population aging, we hoped to have a glimpse of the educational attainment of the working-age population and the health status of older people in Macau, Hong Kong, and Singapore.

**Figure 3:**
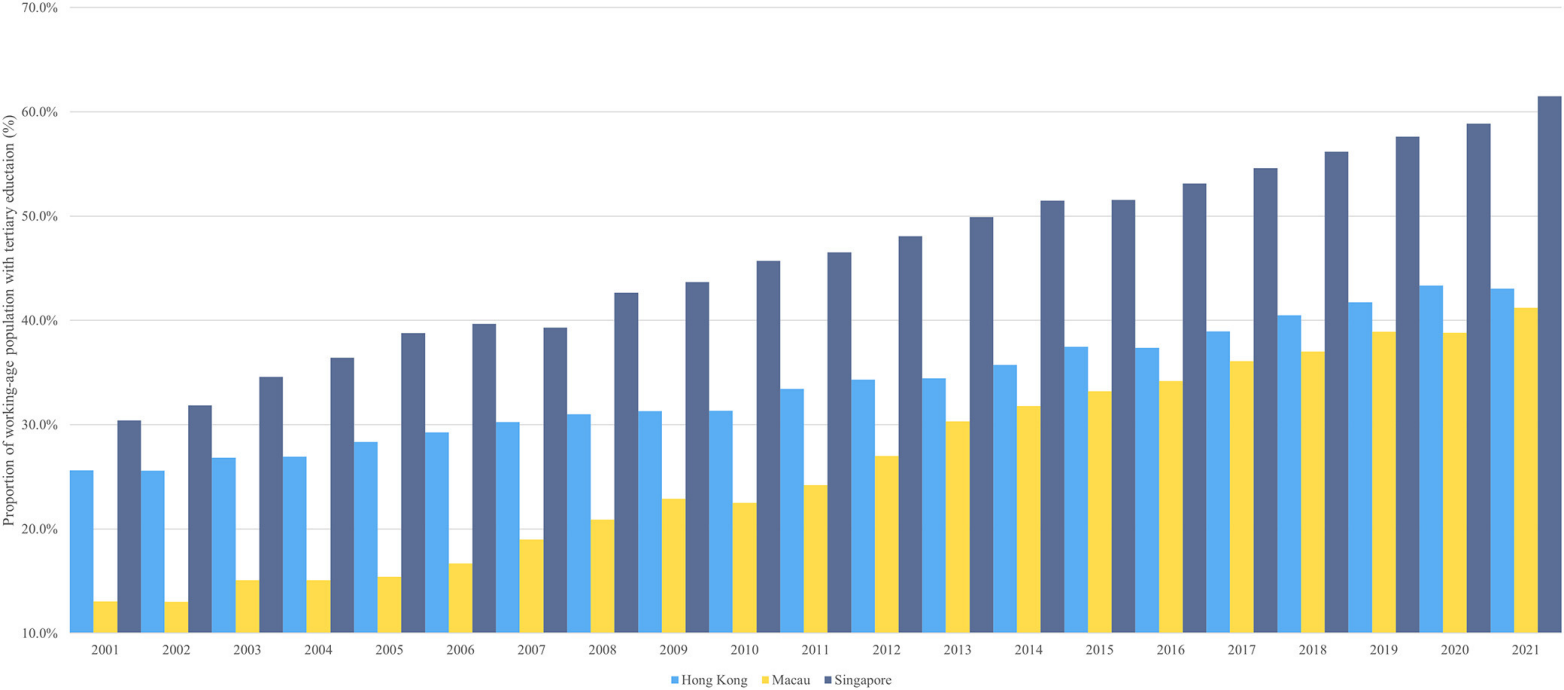
Proportion of working-age people with tertiary education.

Singapore had a considerably higher tertiary education rate for its working-age population than the other two cities, but Macau experienced a surge in growth over past two decades. In 2021, 43.1, 41.2, and 61.5% of the working-age population in Hong Kong, Macau, and Singapore received tertiary education (Figure 3). Singapore led the other two cities in tertiary-educated working-age population from 2001–2021, which doubled from 30.4 to 61.5%. Meanwhile, the proportion of the working-age population in Hong Kong with tertiary education increased steadily, from 25.6% in 2001 to over 40% in 2018. Macau had the lowest proportion of the working-age population with tertiary education, but it grew rapidly from 13.1% in 2001 to 41.2% in 2021, more than tripling in two decades. The gap between Hong Kong and Macau was gradually narrowing down. Hong Kong's working-age population had twice as much tertiary education as Macau in 2001, but only 0.9% by 2021. The gap between Hong Kong's working-age population and Singapore's tertiary education attainment had grown from less than 5% in 2001 to 18.4% in 2021.

**Figure 4:**
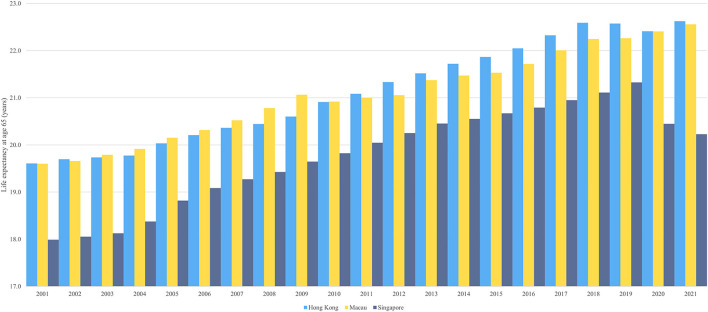
Life expectancy at age 65 in Hong Kong, Macau, and Singapore.

Life expectancy at age 65 measures the overall health of older adults in a region. While all three regions rank among the top in life expectancy at age 65 globally, the figure of Singapore was lower than that of Hong Kong and Macau (Figure 4). In 2021, the life expectancy at age 65 in Hong Kong, Macau, and Singapore was 22.62, 22.56, and 20.23 respectively. Life expectancy at age 65 in Hong Kong and Macau was similar, with the disparity fluctuating within plus or minus 0.5 years. Singapore, on the other hand, had a larger gap with both Hong Kong and Macau, rising to more than 2 years after 2019.

### Aging burden from an education-health adjusted perspective

The Education-Health Adjusted Old-Age Dependency Ratio (EHA-OADR) is an alternative dependency ratio consisting of four sets of formulas, where OADR_h_t_ and OADR_uh_t_ represent the burden of healthy and unhealthy old-age population on the working-age population with tertiary education; similarly, OADR_h_nt_ and OADR_uh_nt_ indicate the burden placed on the working-age population without tertiary education by healthy and unhealthy old-age population. After including the weights for health and education, WEHA-OADR, a combined adjusted dependency ratio, is obtained.

Responding to Q1, there was obvious variations in EHA-OADR values among three cities (Table 2). First, for Macau, we calculated the EHA-OADR in 2004 and 2016, i.e., the 10^th^ and 22^nd^ years after entering aging society. During these 12 years, the OADR_h_nt_ and OADR_uh_nt_ increased by 0.4 and 6.2, while the OADR_h_t_ and OADR_uh_t_ decreased by 13.5 and 15.3. Second, we calculated the EHA-OADR for Hong Kong in 2003 and 2015, i.e., the 20th and 32nd years after the aging society. During this period, only the OADR_uh_t_ fell by 9.4. The OADR_h_nt_ almost doubled, while the OADR_h_t_ and OADR_uh_nt_ both increased by 3.5. Third, we computed the EHA-OADR for Singapore in 2007 and 2011, i.e., the 3rd and 7th years after the aging society. Over the 4 years, the OADR_h_nt_ and OADR_h_t_ increased by 3.8 and 1.0, while OADR_uh_nt_ and OADR_uh_t_ decreased by 1.2 and 3.9.

**Table 2:** The OADR and EHA-OADR for Macau, Hong Kong, and Singapore.

		**Macau**		**Hong Kong**		**Singapore**	
		**2004**	**2016**	**2003**	**2015**	**2007**	**2011**
From the start of the aging society (Years)		10	22	20	32	3	7
Age 15-64	Population	352,190	477,431	4,903,130	5,369,755	3,516,747	4,074,425
	Percent of tertiary-education (%)	15.1	34.2	26.8	37.5	39.3	46.5
Age 65+	Population	33,622	56,160	798,404	1,107,285	329,736	396,310
	Percent of healthy elderly (%)	34.4	23.9	35.8	46.1	57.4	70.1
OADR		9.5	11.8	16.3	20.6	9.4	9.7
EHA-OADR						
OADR_h_nt_		3.9	4.3	8.0	15.2	8.9	12.7
OADR_h_t_		21.7	8.2	21.8	25.3	13.7	14.7
OADR_uh_nt_		7.4	13.6	14.3	17.8	6.6	5.4
OADR_uh_t_		41.5	26.2	39.0	29.6	10.2	6.3

In response to Q2 and Q3, the WEHA-OADR of three cities were all smaller than its conventional OADR (Table 3), but Singapore, with the shortest period of time in an aging society, had the largest decline in WEHA-OADR, followed by Hong Kong and Macau. Meanwhile, as the increase of weights, the WEHA-OADR showed a more substantial reduction. Compared to the conventional OADR, the WEHA-OADR of Singapore reduced by 9.5 to 30.5%, that of Hong Kong declined by 6.2 to 22.5%, and that of Macau dropped by 4.4 to 16.1%. In particular, before weighting, Macau and Singapore have similar conventional OADRs, but after weighting, Singapore's decline in WEHA-OADR is greater. For example, Macau's OADR in 2004 was 9.5 and Singapore's in 2007 was 9.4. After 10% weighting, Macau's dropped by 4.4% while Singapore's declined by 9.5%, more than twice as much as Macau's.

**Table 3:** The comparison of OADR and WEHA-OADR for Macau, Hong Kong, and Singapore.

	**Macau**		**Hong Kong**		**Singapore**	
	**2004**	**2016**	**2003**	**2015**	**2007**	**2011**
From the start of the aging society (Years)	10	22	20	32	3	7
OADR	9.5	11.8	16.3	20.6	9.4	9.7
WEHA-OADR (% of change over OADR)
10% Weight	9.1 (−4.4%)	11.1 (−5.9%)	15.3 (−6.2%)	19.0 (−8.0%)	8.5 (−9.5%)	8.6 (−10.9%)
20% Weight	8.6 (−9.2%)	10.5 (−11.2%)	14.3 (−12.0%)	17.4 (−15.5%)	7.7 (−18.1%)	7.7 (−21.1%)
30% Weight	8.2 (−13.8%)	9.9 (−16.1%)	13.5 (−17.5%)	16.0 (−22.5%)	6.9 (−26.1%)	6.7 (−30.5%)

## Discussion

### Aging burden comparisons from conventional perspectives

Among three cities, Hong Kong encountered with the most serious aging burden, but it performed much better in curbing the growth rate of population aging according to the conventional indicator. Hong Kong has the highest OADR and old-age population proportion, but their growth rates remain relatively low and stable. Consistent with previous studies, this indicates that Hong Kong has been effective in controlling the growth of the old-age population (54, 55). A Five-Pronged Strategy (56) might be the reason why the Hong Kong government has been responding smoothly to the aging population. It includes creating a conducive environment to attract more women and older people into the labor market, increasing vocational and higher education opportunities to promote employment for young people, encouraging labor importation, promoting child-rearing, and building an elderly-friendly environment, of which we will further discuss the education and health-related aspects later.

Three regions all experienced a rapid increase in the growth rate of elderly population proportion and OADR since 2008. This could be explained by the aging of “baby boom” generations after World War II and financial crisis during 2007–2008 (2, 57, 58). First, after 2008, people born during the baby boom after World War II became older adults, causing a sharp increase in the elderly population. Second, in the early 2000s, benefiting from the demographic bonus with low fertility rate and rapid labor force growth, the OADR growth rates of the three regions continued to decline. However, the financial crisis of 2007–2008 triggered a lower or even negative growth in the labor force. As mentioned by Bloom et al. (2), the need for policy adaptations to an aging population will become more important in the face of retirement of the baby boomers and slowing labor force growth. This study shows that the growth of aging and its burden in Hong Kong has remained stable, but those in Macau and Singapore had risen dramatically in the 2010s, suggesting more formidable challenge in Singapore and Macau. For Macau, this may be caused by its single local industry, limited labor market, and strict immigration and labor importation policies. Singapore's problem may originate from a too low total fertility rate.

### Aging burden comparisons from an education-health adjusted perspective

The inclusion of education for the working-age population and health in older people has led to declining in the burden of population aging in Macau, Hong Kong, and Singapore. Responding to Q1, all three regions have different trends in the EHA-OADR. Turning back to Q2, the WEHA-OADRs for Macau, Hong Kong, and Singapore are all smaller than the conventional OADR, and the discrepancy increases with weight. This reveals that in all three regions, the combination of tertiary education for working-age population and health promotion for older adults is effective in mitigating the burden of aging. However, contrary to Q3, longer years into an aging society does not imply a lower WEHA-OADR.

#### Macau

From 2004 to 2016, increase in OADR_h_nt_ and OADR_uh_nt_ and reduction in OADR_h_t_ and OADR_uh_t_ was observed in Macau, which might be related with the improvement in education among labor force. In a previous study, the EHA-OADR in Mainland China showed a similar trend, which is also explained by the rapid progress of higher education (46). The overall health rate of the elderly in Macau is not high even declining, but the tertiary education rate in the working-age population surges. Before 1999, the Portuguese colonial government did not value higher education in Macau. Gaming as monolithic economic background also made Macau society less focused on higher education. Therefore, in the early 21^st^ century, Macau's working-age population had a lower tertiary education rate than Hong Kong and Singapore. After Macau's return to China in 1999, the booming economy provided more funds to develop higher education. In primary education, the government offers 15 years of free education, the longest of the three regions. Also, more people are getting tertiary education as universities expand (59, 60).

As shown, the difference between WEHA-OADR and OADR in Macau is the smallest comparing with Hong Kong and Singapore, which could be explained by the relatively poor health status of elderly population in Macau. Different from Hong Kong and Singapore, Macau is smaller and more densely populated, which makes the allocation of medical resources more challenging. Table 2 shows that while tertiary education in the working-age population doubled between 2004 and 2016, the percentage of healthy elderly decreased from 34.4 to 23.9%. Regardless of numerous attempts from government, the sharply growth of elderly population and limited lands impede the progress toward improving elderly groups' health status in Macau (61). First, residents aged 65 and over are exempt from all medical fees at the public hospital. For some patients who cannot be treated in Macau, the government will provide outbound medical services. Second, since the 1980s introduction of primary healthcare, a comprehensive network of medical services provides primary care for the elderly. Seniors can also use government-issued medical vouchers at over 600 private clinics. Third, the Macau government prioritizes elderly community care. Macau's government currently subsidizes 18 elderly homes and three District Elderly Community Centers. However, there is only one public hospital in Macau. Whether it is a visit to the public hospital or a stay at an elderly home, older people usually have to make appointments weeks or even months in advance. Medical vouchers may not cover long-term follow-up care for older people with chronic conditions (62). In recent years, the COVID-19 pandemic has hindered outbound medical service for some patients. In general, Macau's public health care system seems to be comprehensive, but in fact the coverage for the elderly is not very high. Extremely limited space and medical resources may make it difficult for Macau to cope with the increasing elderly population in the future. Expanding medical and care services in Guangdong-Hong Kong-Macau Greater Bay Area is believed to be a promising solution for Macau (61).

#### Hong Kong

In Hong Kong, only the OADR_uh_t_ fell and the other three EHA-OADRs increased, this pattern has been discussed in a previous study referring to Japan (46). In regions with a prolonged aging process, like Hong Kong and Japan, improved healthcare has increased life expectancy. As more and more older people, especially healthy ones, can live longer, resulting in the rise of the OADR_h_t_, OADR_h_nt_, and OADR_uh_nt_. To enhance capacity to support older people, Hong Kong government further boosts its education. One of the Five-Pronged Strategy is increasing vocational and higher education opportunities. The Hong Kong government has increased the number and enrolment of full-time and part-time higher education, vocational education, and sub-degree programs, and provided financial assistance to some students, thus contributing to the tertiary education rate.

However, the previous accumulated health care resources are not enough to cope with the increasingly serious aging population, which is the reason why Hong Kong's WEHA-OADR has not dropped as much as Singapore's. Over last 170 years, Hong Kong has developed a two-tiered system of government-led and market-supported healthcare (63). The public health system in Hong Kong is partly similar to the British National Health System, about 90% of inpatient services are provided by the public hospital system. The private system, on the other hand, offers about 70% of all fee-for-service outpatient services. Hong Kong faces multiple challenges in its healthcare system, with a heavy workload of public clinical staff and an overstretched public hospital system that results in long waiting times for non-emergency procedures (64). Under current policy, Hong Kong may have trouble covering the adults' medical costs in the future. As ST Cheng et al. refer to, elder poverty and rebalancing the long-term care (LTC) system are two key aging policy issues in Hong Kong (54). Unlike Singapore's advance-prepared pension system, Hong Kong does not have a public pension system based on tax revenue. Although the government launched the Mandatory Provident Fund Scheme in 2000, which is a personal retirement account with contributions from full-time employees and employers. It will take many years to take effect. For aging baby boomers who have not saved so far, it is too late to accumulate sufficient funds. In 2019, the Hong Kong government implemented that Voluntary Health Insurance Scheme (VHIS) to relieve the pressure on the public healthcare system in the long run. The VHIS is a voluntary and government-regulated private medical insurance program. However, according to previous studies (65, 66), comparing to the health insurance system in Singapore, the intrinsic shortcoming of the VHIS is adverse selection that may paralyze its finance, and as people continue to rely on public medical system, this moderate health care financing reform can hardly achieve its goals. On the other hand, the imbalanced LTC system in Hong Kong depends heavily on residential care. The residential care placement is very limited, and there are many problems with the service quality provided by private residential care homes (67). The Hong Kong government needs to develop a sustainable LTC policy to facilitate the development of home or community care services, and to establish a viable financing mechanism for LTC services.

#### Singapore

From the results of Singapore, we find a pattern of EHA-OADR that has not been seen in previous studies: the OADR_h_nt_ and OADR_h_t_ increased while the OADR_uh_nt_ and OADR_uh_t_ decreased, which mainly concerned with improvements in senior health. Singapore's elderly health rate was initially higher than Hong Kong and Macau, with 57.4% in 2007, the third year of its aging society. And it only took 4 years for the rate to reach up to 70.1%. This is due to Singapore's excellent health system, which was ranked 6th by the WHO in 2000 (68). Apart from government subsidies, there are three main healthcare financing approaches in Singapore: (1) MediSave: Compulsory individual medical savings account to which Singaporeans contribute around 8.0–10.5% of their wages; (2) MediShield Life (evolved from MediShield before 2015): Mandatory lifelong basic insurance plan that caters for large hospital bills and selected costly outpatient treatments; (3) MediFund: Endowment fund set up by the Government as a safety net for Singaporeans who face difficulty to pay medical bills. This government-and-citizens-shared health care system funds the elderly's health care in advance (69).

Referring to previous research (46), we assumed that regions that have been in the aging society longer tend to have better education and health resources, and thus have greater declines in WEHA-OADR. Yet, Singapore, the youngest aging society, has the largest decline in WEHA-OADR, followed by Hong Kong and Macau. The results suggest that even with a short time in an aging society, WEHA-OADR can drop significantly. What is Singapore's secret? Well-prepared health and education. We have talked about the strengths of Singapore's health system, and what about education? Dating back to the early days of Singapore's establishment, small land size and lack of natural resources make labor Singapore's primary resource. Singapore's government needed a well-trained labor force from the 1960s to the 1980s to attract foreign investment. The urgency for education led to a massive school building, curriculum standardization, and teacher training centralization (70). In 1990, the tertiary education rate for Singaporeans aged 25 and above had reached 8.4% (71). As the economy matured and skill requirements changed, Singapore's education became more diverse to foster different talents with tertiary degree. In short, education has always been an essential development strategy for Singapore (41). The early accumulation of higher education talent provided Singapore with more dependency at the beginning of an aging society. Recently, taking into account its aging population, Singapore has increasingly relied on international students for foreign talent (72).

### Comparative analysis from political proposition

Increasing the higher education rate of the working-age population improves society's ability to sustain the elderly, while enhancing older people's health decreases their burden on society. With a more educated workforce, more workers can join innovative industries, boosting the market's productivity. Better quality human resources can also attract investment. Moreover, a healthier old-age population eases medical and long-term care needs, which can reduce the pressure on government spending on healthcare. In this study, the WEHA-OADR is smaller than the conventional OADR in the three cities. Macau, Hong Kong, and Singapore should therefore consider the education of working-age population and elderly health when formulating their population policies.

Looking back at the population policies in the past two decades, we find Hong Kong's population policy focuses on immigration and talent recruitment to rejuvenate its age structure. As early as 2003, in the Report of the Task Force on Population Policy, the Hong Kong government proposed “Admission of Mainland Professionals and Talent” and “Investment Immigrants” (73). By 2015, the Five-Pronged Strategy mentioned “Adopting a more proactive and targeted approach to attract more outside talent to work and settle in Hong Kong to build up our stock of human capital. We should also consider more effective importation of labor arrangements for industries suffering from persistent manpower shortage without jeopardizing the interests of local workers” (56). At present, Quality Migrant Admission Scheme, General Employment Policy, and Admission Scheme for Mainland Talents and Professionals are Hong Kong's three major talent admission policies. In 2010, Hong Kong's net in-migration had reached 16,700 people and is expected to reach 39,600 by 2050 (74).

Due to its limited geographical size, Macau is less driven to bring in talent and encourage immigration than Hong Kong, preferring to boost local education. Since 2011, the Macau government has proposed the “Macau Thrives on Education” and “Building Macau through Talent Training” policies (75). The “Macau Thrives on Education” has given Macau students a better environment for further education. In 2015, Macau's education budget was 11.7%, similar to the OECD average. Meanwhile, the Macau government has added free education allowance, tuition fee allowance, and book allowance. The “Building Macau through Talent Training” further enhances the quality and competitiveness of Macau's working-age population. In 2014, the Macau government established the Talent Development Committee and began a program to finance excellent candidates for master's degree programs and short-term training.

Like Hong Kong, Singapore has established strategies to encourage immigration. From the 1990s until 2010, Singapore liberalized its immigration policy and enabled all enterprises to hire up to 50% foreigners. After 2010, Singapore tightened its immigration policies and gave social support to existing immigrants to ensure their effective assimilation. Notably, Singapore is the only one of the three regions that explicitly encourages childbirth. Singapore promotes posters to encourage conception and hosts annual social gatherings for singles. It has also engaged in dozens of conversations with the public on fertility issues in national speeches (76). In contrast, in the public consultation in 2012, only 40% of Macau people supported the idea of “adopting a fertility policy to cope with aging”, so it is not advisable for Macau to strongly pursue a fertility policy (75). Also, childbirth promotion requires government funding. Singapore introduced a housing scheme to help married couples build homes, a baby bonus scheme and a Child Development Account (CDA) to help parents raise their children and provided up to 16 weeks of maternity leave for mothers and 7 days of paternity leave for fathers after the birth of their babies. A study demonstrates that the Hong Kong government avoids the financial commitment of childbirth, which prevents childbirth from improving (77).

Improving the education of the working-age population and the health of the elderly are both beneficial for Hong Kong and Macau, provided that fertility cannot be increased in the short run. Hong Kong can optimize its talent admission scheme and encourage the return of emigrants. Also, the Hong Kong government should seek sustainable health financing and long-term care options. Macau can increase subsidies for higher education through publicly sponsored and self-financed institutions and enhance primary education to accommodate its population density and boost the future job market. Meanwhile, Macau can extend its medical and care services in the Guangdong-Hong Kong-Macau Greater Bay Area to overcome its limited area and medical resources. Singapore may create alternative strategies to boost fertility while retaining education and health policy.

## Conclusions

This is the first study to compare the burden of population aging combining the education of working-age population and the health of old-age population in Macau, Hong Kong, and Singapore. After including education and health characteristics, the burden of population aging in the three cities decreases, with the highest reduction in Singapore and the smallest in Macau, suggesting that boosting working-age population education and older health can help reduce population aging burden. Hong Kong may pursue sustainable health funding and long-term care options to deal with its aging population. Macau should increase subsidies for both higher and primary education, as well as expand its medical and care service externally. Singapore may seek further incentives to encourage fertility.

There are some limitations in this study. First, due to a lack of data on the self-rated health results, for each region, we only calculated the EHA-OADR and WEHA-OADR for 2 years. The selected years may not be conducive to a longitudinal comparison, but they represent different stages of aging in the three regions, allowing interregional comparisons. Second, Macau's self-rated health results came from 60-and-over samples and Singapore's 2007 data came from 60-to-69-year-olds. Since older groups tend to be less healthy, we may overestimate Macau's elderly health rate. And because Singapore's 2011 data are from those 65 and older, we may have underestimated Singapore's progress in senior health. To deal with the above limitations, other unified parameters may be adopted. For example, the prevalence of regular exercise (46), functional impairment (78) and aging-related disease burden (5). Lastly, this study is retrospective and makes no projections. Follow-up studies can make projections by using existing data from other regions as forecast data (46), developing linear equations (12), or obtaining forecast data from other sources (79).

## Data availability statement

The original contributions presented in the study are included in the article/supplementary material, further inquiries can be directed to the corresponding author/s.

## Author contributions

D-mX: conceptualization, investigation, and writing—original draft. QB: writing—review and editing. YB: conceptualization, supervision, and writing—review and editing. All authors contributed to the article and approved the submitted version.

## Funding

This study was supported by The Science and Technology Development Fund, Macau SAR (SKL-QRCM(UM)-2020-2022) and University of Macau research funding: Project Ref No. QRCM-IRG2022-001.

## Conflict of interest

The authors declare that the research was conducted in the absence of any commercial or financial relationships that could be construed as a potential conflict of interest.

## Publisher's note

All claims expressed in this article are solely those of the authors and do not necessarily represent those of their affiliated organizations, or those of the publisher, the editors and the reviewers. Any product that may be evaluated in this article, or claim that may be made by its manufacturer, is not guaranteed or endorsed by the publisher.
